# Correction to ‘Learning earth system models from observations: machine learning or data assimilation?’

**DOI:** 10.1098/rsta.2022.0004

**Published:** 2022-04-04

**Authors:** A. J. Geer

*Phil. Trans. R. Soc. A* **379**, 20200089. (Published online 15 February 2021). (doi:10.1098/rsta.2020.0089)

In this article, there was an error in equation 2.5, as the logarithm should have been multiplied by −2, not −1. This has now been corrected.

